# What If Trojan Horse Nanoparticles Could Change the Game for HPV Gene‐Targeted Therapies?

**DOI:** 10.1002/jmv.70916

**Published:** 2026-04-13

**Authors:** Trairong Chokwassanasakulkit, Vindya Ranasinghe, Erin Woods, Linh Q. Nguyen, Nigel A. J. McMillan

**Affiliations:** ^1^ Institute of Biomedicine and Glycomics and School and Pharmacy and Medical Sciences Griffith University Gold Coast Queensland Australia

**Keywords:** cervical cancer, CRISPR/Cas, gene‐targeted therapy, human papillomavirus (HPV), siRNA, Trojan horse nanoparticles

## Abstract

Human papillomavirus (HPV) is a common sexually transmitted infection linked to various cancers, particularly cervical cancer, primarily driven by high‐risk strains like HPV16 and HPV18. While vaccines are effective in preventing new infections, they do not address existing cases, highlighting the need for innovative therapies. Gene‐targeted approaches, such as CRISPR/Cas and siRNA, show promise in inhibiting HPV oncogenes. Recent advancements in Trojan horse nanoparticles (NPs) offer a strategy for delivering these therapies directly to HPV‐infected cells. These NPs improve stability and targeted delivery, enhancing the biodistribution of CRISPR/Cas systems and siRNAs while protecting them from degradation. However, challenges like immune responses and regulatory hurdles persist. Therefore, this review emphasizes the potential of Trojan horse NPs in treating HPV‐related cancers, identifies critical areas for future research, and provides updates on gene‐targeted therapy encapsulated NPs in preclinical and clinical trials.

## 1 Introduction

Human papillomavirus (HPV) is one of the most prevalent and impactful viral pathogens associated with human disease. HPV is notably recognized as the most prevalent sexually transmitted infection globally, leading to a significant public health burden. High‐risk HPV types, particularly HPV16 and HPV18, are responsible for approximately 70% of cervical cancer cases, which account for nearly 5% of all cancer diagnoses and fatalities worldwide [1]. This staggering statistic underscores the necessity for enhanced effective preventive and therapeutic strategies against HPV‐related malignancies.

Despite the availability of effective HPV vaccines, access to vaccination remains inequitable, particularly in low‐ and middle‐income countries (LMIC) where cervical cancer rates are disproportionately high. Barriers such as socioeconomic disparities and healthcare infrastructure limitations hinder the widespread implementation of preventive measures, leaving many individuals at risk for persistent HPV infections that can lead to oncogenic transformations [2]. Current treatments for HPV‐related cancers predominantly involve surgical intervention alongside chemoradiation; however, these approaches have not significantly improved the outcome and survival rates, underscoring the necessity for novel therapeutic avenues, specifically gene‐targeted therapy [3].

Gene‐targeted therapy for HPV represents a promising frontier in the treatment of HPV‐associated diseases, particularly cervical cancer. This approach utilizes advanced techniques such as Clustered Regularly Interspaced Short Palindromic Repeats (CRISPR/Cas) systems and small interfering RNA (siRNA) to specifically target and disrupt HPV oncogenes, which play a crucial role in cancer development. CRISPR is a specific gene editing technology utilized to induce double‐strand breaks in DNA using the Cas9 protein. The CRISPR/Cas technology, particularly when delivered in nanoparticle formulations, enhances the stability and bioavailability of gene‐editing components, ensuring targeted delivery to affected cervical cells, thereby reducing tumor size and HPV gene expression in preclinical models [4, 5, 6]. Meanwhile, siRNA is a small double‐stranded RNA molecule that silences specific genes by degrading their target mRNA, preventing translation into protein. Although, siRNA therapies are potent in silencing genes, they face significant challenges including instability in circulation and difficulties in cellular uptake due to their hydrophilic nature [7, 8]. Ongoing research is essential to overcome these barriers and improve delivery systems, ultimately paving the way for effective clinical applications of gene‐targeted therapies for HPV‐related conditions. Notably, studies have shown that HPV‐positive patients generally respond more effectively to treatments because the presence of viral antigens makes the cancer more immunogenic, highlighting the urgent need for innovations in delivery systems that can amplify these therapeutic outcomes [9].

The revolutionary approach of utilizing Trojan horse NPs in gene therapy offers a game‐changing solution for enhancing the efficacy of HPV treatment by significantly improving delivery methods. These nanoparticles can encapsulate therapeutic nucleic acids within protective carriers that are specifically functionalized to target HPV‐infected cells, thus optimizing biodistribution and reducing systemic toxicity [10]. By leveraging receptor‐mediated transcytosis (RMT), these advanced particles can efficiently traverse physiological barriers like the blood‐brain barrier (BBB), ensuring that therapeutic agents accurately reach their intended targets with unparalleled precision [11]. As advancements in nanotechnology continue to emerge, the integration of these innovative delivery systems into HPV gene targeted treatments holds the promise to revolutionize treatment paradigms. Therefore, this comprehensive review will emphasize these Trojan horse nanoparticles, aiming to unveil new and effective methods in the fight against HPV‐related diseases, while also providing updates on state‐of‐the‐art gene‐targeted therapies, including RNA interference (RNAi) and the CRISPR/Cas system.

## 2 HPV Mechanisms and Gene‐Targeted Strategies

HPV is a small double‐stranded DNA virus that belongs to the *papillomaviridae* family. It is the most frequently encountered sexually transmitted disease, and transmission occurs through direct contact between the skin and the mucous membrane [3]. HPV is broadly classified into two types, cutaneous and mucosal, based on the tissue tropism. Mucosal HPV is further classified into low‐risk and high‐risk HPV based on the neoplastic progression of the lesions. High‐risk HPV is accountable for around 5% of all human cancers, and almost all cases of cervical cancers are attributed to high‐risk HPV types [1]. HPV consists of three functional regions in its genome; the second is the “early region.” The early region is responsible for the viral replication and carcinogenesis process as it encodes *E1*, *E2*, *E4*, *E5*, *E6*, and *E7* viral oncoproteins [9]. HPV induced cancers are dependent on the expression of *E6* and *E7* viral oncogenes. Therefore, many therapeutic interventions are centered on inhibiting the expression of these viral oncogenes [12].

High‐risk HPV types, HPV16 and HPV18 are accountable for approximately 70% of cervical cancer incidents. The HPV oncogenes *E6* and *E7* inhibit the function of *p53* and *pRb*, respectively, resulting in uncontrolled cell proliferation. In most patients, the infection gets cleared off by the immune system. However, a persistent infection can lead to genetic alterations in the cells and ultimately result in neoplastic lesions [13]. Even though preventive vaccines are available, they are not effective for established HPV infections. Moreover, low socioeconomic countries find it difficult to afford preventive vaccination programmes. Hence, it is prudent to discover novel therapeutic strategies [2]. Currently, the treatment of cervical cancer is surgery combined with chemoradiation. The management is decided considering the stage of the disease. For the early stage of disease, radical hysterectomy is indicated along with pelvic lymphadenectomy. For locally advanced disease and metastatic disease, chemoradiotherapy is recommended [14].

In addition to cervical cancer HPV is also associated with other malignancies such as oropharyngeal, anal, vaginal, penile and vulvar carcinomas. There is a well‐established association between oropharyngeal squamous cell carcinoma (OPSCC) and HPV infection [15]. HPV infection was positive in approximately 35.6% of OPSCC cases, and HPV16 being the predominant genotype. The treatment option is surgery with chemoradiation therapy [16]. Vulval carcinomas are commonly found in menopausal women, and based on the presence of HPV infection, they are categorized into HPV‐associated and HPV‐independent groups. Interestingly, HPV positive vulvar cancer patients respond better to radiotherapy compared to HPV‐negative counterparts [17]. Vaginal cancers are also diagnosed in older women who attained menopause. However, the presence of high‐risk HPV infection can initiate the cancers even in younger women [18]. HPV associated and HPV independent penile cancers are distinguished histologically based on morphological differences. Around 50% of penile cancers are HPV positive, and HPV16 is the most frequently found genotype. It is known that the disease‐specific survival is higher in the HPV‐positive penile cancer patients compared with HPV negative patients [18]. Correspondingly, the survival rates of anal cancer patients are higher in the HPV positive cases compared to HPV negative cases [19].

Existing therapy for HPV positive cancer has not increased the outcome and survival of patients in recent years, and there is a crucial need for novel therapeutic strategies. Gene therapy is currently studied extensively as a potential strategy for cancer regression. Gene therapy is defined as the modification of a patient's cells at the genetic level to treat a disease that does not respond to established medical therapies [20]. Gene editing technology is emerging as a promising gene therapy in cancer as it can adjust and correct the expression of mutated genes. CRISPR is more popular than other gene editing technologies (zinc finger endonuclease and transcription activator‐like effector nuclease) due to its simplicity, reliability, and low cost [21]. CRISPR is known to have the highest potential for genome editing compared to other gene editing tools.

Even though many types of CRISPR/Cas systems are available, CRISPR Cas9 is the most studied and well‐described CRISPR system [22]. The CRISPR/Cas9 system was first described in prokaryotes as a mode of adaptive immunity to bacteriophages and some infections. It consists of two components: Cas9 endonuclease and single‐stranded guide RNA (sgRNA). The sgRNA guides the Cas9 endonuclease enzyme to the target gene, where the double‐strand break occurs, resulting in genomic modifications [21]. CRISPR/Cas9 has several variants, namely CRISPR activation (CRISPRa) and CRISPR interference (CRISPRi), which can cause alterations in gene expression without any modifications to the sequence [23]. The major drawback of CRISPR/Cas9 technology is the off‐target effect. Hence, it is of great importance to minimize the off‐target effects to use CRISPR as a safe therapeutic approach [24].

Another strategy in gene therapy is RNAi technology, which involves the use of complementary short RNAs to inhibit the expression of specific genes [25]. Antisense RNA, siRNA, and short hairpin RNA (shRNA) are synthetic RNAi methods. SiRNA and shRNA are more frequently used as therapeutic strategies in HPV gene therapy. Each of the two shares a similar mechanism of function in the gene silencing process. Both form the RNA‐Induced Silencing Complex (RISC) after entering the cytoplasm and initiate the mRNA degradation and inhibition of translation. However, shRNA molecules are degraded by the dicer protein before making the RISC complex [26].

**Figure 1:**
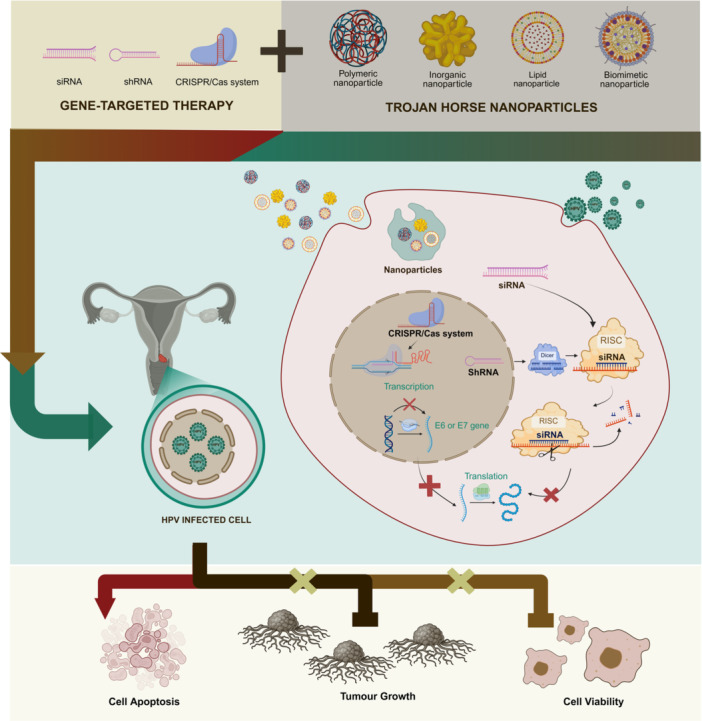
Schematic representation of the integration of gene‐targeting instruments (siRNA, shRNA, and CRISPR/Cas) with Trojan horse nanoparticles to enhance delivery into HPV‐infected cells. Polymeric, inorganic, lipid, and biomimetic nanoparticles safeguard and convey nucleic acids, facilitating the silence or disruption of HPV E6/E7 oncogenes. Improved intracellular delivery facilitates apoptosis, inhibits tumor proliferation, and diminishes cell viability, highlighting that therapeutic efficacy relies not only on the accuracy of gene editing but also on effective targeted delivery.

The pursuit for effective gene‐targeted therapy hinges not just on the precision of gene editing but, crucially, on the delivery mechanisms that transport therapeutic nucleic acids to their intended cellular targets (Figure 1). However, delivery has many limitations, including degradation of nucleic acids by hydrolysis, poor cellular uptake, and the negatively charged nature of nucleic acids. As a solution, researchers employ vectors to make the delivery process more efficient. Non‐viral vector‐based delivery systems are becoming the focus of current studies. Trojan horse NPs, which include lipid nanoparticles (LNPs), polymeric nanoparticles, inorganic nanoparticles and biomimetic nanoparticles, are used in non‐viral delivery methods, with LNPs being the most employed [27].

## 3 Trojan Horse Nanoparticles in Gene Therapy

Nanoparticles engineered as “Trojan horses” are advanced drug delivery systems designed to mimic biological entities or exploit endogenous transport pathways to bypass physiological barriers such as the BBB and cell membranes. The term draws analogy from the ancient Greek narrative, where these NPs disguise therapeutic nucleic acids (e.g., plasmid DNA, siRNA, mRNA, or CRISPR‐Cas systems) within protective nanocarriers. These carriers are functionalized with targeting ligands (e.g., monoclonal antibodies [mAbs], peptides) or cloaked with biomimetic coatings (e.g., cancer cell or immune cell membranes) to evade immune detection and navigate biological obstacles [11, 28]. This approach addresses critical challenges in gene therapy, including poor biodistribution, rapid clearance, and off‐target effects. For instance, while viral vectors like Adeno‐Associated Viruses (AAVs) face cargo limitations (< 4.7 kb) and immunogenicity, Trojan horse NPs offer modularity, scalability, and reduced immunogenic risks [11].

Nanoparticles can encapsulate therapeutic agents, such as siRNA, which can knockdown HPV genes, ensuring that the treatment is delivered specifically to HPV‐infected cells [29]. The nanoparticle coated with ligands can bind to HPV capsid proteins, disrupting their structure and preventing viral entry into host cells [30]. Utilizing cancer cell membranes as a coating for nanoparticles can disguise them from the immune system, facilitating their uptake by antigen‐presenting cells (APCs) and enhancing the immune response against HPV‐infected cells [31].

Based on composition and functionalization, the Trojan horse NPs are classified to: LNPs, Polymeric nanoparticles, Inorganic nanoparticles and Biomimetic nanoparticles.

### 3.1 Lipid Nanoparticles

LNPs exemplified by their pivotal role in COVID‐19 mRNA vaccines, are composed of ionizable lipid, cholesterol, and PEGylated phospholipids [32]. These cationic lipids components enable efficient encapsulation and protection of nucleic acids, such as mRNA or plasmid DNA, while minimizing immunogenicity [33]. Polyethylene glycol (PEG), a hydrophilic polymer, is widely used to modify the LNP's surface. This modification has several benefits to help overcome the biological obstacles of LNPs. Firstly, PEG‐lipids prevent aggregation and stabilize the particles. PEG provides the steric barrier at the surface of LNPs, so they can prevent the particle fusion and formulate a homogenous population of LNPs [34]. Secondly, the PEGylation significantly extends the system circulation of LNPs so enhance the pharmacokinetics and effectiveness [35]. PEGylated lipids enhance the blood circulation time and protect the LNPs surface, decreasing the kidney's clearance. PEGylated lipids also protect LNPs from uptake by the Mononuclear Phagocyte System (MPS) [36]. Thirdly, the PEGylation reduces the immunogenicity of LNPs, making them less to be recognized and cleared by the immune system [37]. Surface functionalization with ligands, such as mAbs targeting the transferrin receptor (TfR), allows LNPs to exploit endogenous transport pathways. For instance, TfR‐targeted LNPs were more effective than non‐targeted LNPs and free G3139, antisense oligonucleotide, in decreasing 62% of Bcl‐2 mRNA expression and in inducing caspase‐dependent apoptosis [38]. The conjugation of TfR to the surface of the LNPs enables RMT across the BBB and subsequent localization to the nucleus for transcription of the therapeutic gene [11]. In cancer treatment, RMT is exploited in nanomedicine to enhance the penetration of anti‐cancer drugs into solid tumors, which are otherwise difficult to reach through passive diffusion [39]. Jing et al. [40] reported that TfR is also significantly overexpressed in cervical cancer compared to normal tissues. Because it is efficiently internalized, TfR is a primary target for delivering nanoparticles and therapeutic genes directly into malignant cells [41]. Beyond gene delivery, LNPs have been repurposed as carriers for gold NPs (GNPs) in radiotherapy, enhancing GNP uptake in triple‐negative breast cancer cells by 73‐fold and amplifying radiation‐induced DNA damage [42].

### 3.2 Polymeric Nanoparticles

Polymeric nanoparticles, such as those composed of poly(lactic‐co‐glycolic acid) (PLGA), poly (β‐amino ester) (PBAE)), leverage biodegradability and tuneable surface properties for targeted delivery [43]. PLGA leads to a prolonged drug release profile, decreasing harmful side effects and improving patient compliance. Zhang et al. [44] synthesized two curcumin (CUR) delivery systems for Alzheimer's disease: (i) chitosan (CS)‐coated PLGA nanoparticles encapsulating CUR (CUR‐CS‐PLGA‐NPs) and (ii) hydroxypropyl‐β‐cyclodextrin (HP‐β‐CD)‐based CUR inclusion complexes (CUR/HP‐β‐CD). Both formulations retained structural integrity and drug‐loading efficiency for over 60 days [45] and reduced cytotoxicity compared to free CUR while exhibiting potent antioxidant and anti‐inflammatory activity [44]. Another group also developed PLGA nanoparticles encapsulating the antigenic peptide HPV16 *E7*, utilizing ATP as a new adjuvant, enhancing antigen presentation [46]. Loading doxorubicin in PLGA nanoparticles enabled brain delivery [47]. Cisplatin is an established first‐line chemotherapeutic agent for cervical cancer, as previously documented [48]. To mitigate associated drug resistance and systemic toxicity, Dana et al. [49] developed a cisplatin‐loaded nanoparticle system utilizing liposomes and poly(lactic‐co‐glycolic acid) (PLGA). This system, designated L‐PLGA‐Cis‐Avastin, was fabricated via a double emulsion solvent evaporation method, with the anti‐angiogenic monoclonal antibody bevacizumab (Avastin) conjugated to the lipid component. Evaluations employing 3D spheroid cultures and xenograft models demonstrated that the L‐PLGA‐Cis‐Avastin system exhibited enhanced cellular uptake efficiency and superior binding capability compared to relevant controls. Additionally, silver‐based nanocarriers improved tumor‐specific toxicity in HeLa cells [50].

### 3.3 Inorganic Nanoparticles

Inorganic nanoparticles exhibit inherent physicochemical properties—including magnetic responsiveness, thermal stability, optical tunability, and catalytic activity—serving as robust structural platforms for integrating multiple dopant ions to engineer multifunctional nanocomposites with programmable biomedical functionalities [51]. Inorganic nanoparticles demonstrate superior drug‐loading efficiency, enhanced physicochemical stability (e.g., resistance to enzymatic/oxidative degradation), and precisely tunable degradation kinetics compared to organic nanoparticle counterparts, enabling controlled therapeutic payload release in biologically relevant environments [52]. Recent research focus on the development in the application of gold nanoparticles (AuNPs), silver nanoparticles, graphene‐based nanomaterials, iron oxide, zinc oxide, hydroxyapatite, and cerium oxide nanoparticles for efficient drug delivery. Saifullah et al. [53] designed a nanocarrier that comprises zinc layered hydroxide and PAS (para‐aminosalicylic acid). According to the author, the developed material exhibited quadruple efficiency, in comparison with the pure PAS against *M. tuberculosis* [53]. Feng et al. [54] designed dynamic switching enabled‐gold nanospheres of size ~80 nm. These nanospheres exhibited high penetrability, reduced toxicity, and pH‐dependent drug release. This nanoparticle enhanced brain tumor targeting [54]. Johnsen et al. [55] evaluated the receptor‐mediated uptake of TfR‐targeted antibody‐conjugated AuNPs at the brain capillary endothelium. Their results demonstrated that cellular internalization efficiency was valency‐dependent, with monovalent antibody‐functionalized AuNPs exhibiting enhanced uptake compared to multivalent counterparts. Furthermore, AuNPs conjugated with low‐affinity antibodies facilitated transient brain endothelial accumulation, while high‐affinity binding paradoxically reduced cellular internalization due to potential receptor saturation or impaired endocytic recycling [55]. Gold particles packed with gallic acid reduced tumors without hurting normal cells [56], induce the cervical cancer cell apoptosis by activating caspase 3/7,8, and 9 [57] while gold particles combined with doxorubicin were more effective in attacking cervical cancer cells with fewer side effects [58]. Furthermore, the study showed that Selenium nanoparticles (SeNPs) have the potential to inhibit the proliferation of the cervical cancer cells as well as reduce the angiogenic activity [59].

### 3.4 Biomimic Nanoparticles

The cell membrane serves as a fundamental structural and functional entity, orchestrating critical processes such as ligand‐receptor signaling, immune recognition, and transmembrane signal transduction through its dynamic lipid bilayer architecture and embedded protein networks. This compartmentalization system enables selective permeability, molecular transport, and intercellular communication, with membrane fluidity and protein‐lipid interactions directly modulating these activities [60]. The biomimetic coating of NPs with native cell membranes via direct membrane transfer preserves membrane‐bound proteins, lipids, and glycans, conferring bifunctional properties on the NP platform that mirror the source cell's intrinsic capabilities, such as immune evasion and tissue‐specific homing [61]. Extensive research has demonstrated the utility of cell membrane‐derived nanocoating strategies, harnessing diverse biological membranes—such as erythrocyte, cancer cell, leukocyte, stem cell, bacterial outer membrane, and platelet sources—to functionalize NP surfaces. The study shows that B16F10 tumor cell membranes were coated with CpG‐loaded aluminum phosphate nanoparticles (APMC), enhancing tumor antigen uptake by DCs and promoting powerful immunity [62]. Cell membrane‐coated nanoparticles (CMCNPs) exhibit superior co‐delivery capacity for multi‐agent therapeutics. CCM‐PLGA‐NPs were engineered to co‐deliver paclitaxel and siRNA targeting the HPV E7 oncogene, enhancing tumor‐targeted delivery and promoting apoptosis [63].

## 4 Gene Therapy Nanoparticles for HPV: Status Update

### 4.1 Preclinical Trials

#### 4.1.1 CRISPR/Cas System

The use of CRISPR/Cas encapsulated within Trojan horse‐NPs represents a significant advancement over naked CRISPR/Cas systems in gene therapy for HPV‐related diseases. Encapsulation enhances the stability and bioavailability of the CRISPR components during systemic circulation. Naked CRISPR/Cas is vulnerable to degradation by nucleases, resulting in a shorter circulation half‐life, which leads to decreased efficacy and delivery challenges [4]. Conversely, LNPs, such as cationic liposomes and polymers, offer protective barriers that shield the CRISPR machinery, facilitating its targeted delivery to cervical cancer cells expressing HPV oncogenes. This encapsulation not only improves cellular uptake but also enhances the likelihood of successful gene editing, resulting in more efficient and sustained therapeutic outcomes [5, 6]. To date, only CRISPR/Cas9 has been tested in preclinical trials, along with one study involving CRISPR/shRNA (Table 1).

**Table 1:** Overview of CRISPR/Cas systems utilizing Trojan horse‐like nanoparticles for HPV in preclinical trials.

CRISPR/Cas	Target gene	Nanoparticle		Mechanism of nanoparticles	Therapeutic efficacy	Ref.
		Material	Properties			
CRISPR/Cas9	HPV16 *E6*/*E7*	LNP	Nanoparticle cargo dose: 60 μg (high), 40 μg (medium), and 20 μg (low)	Liposomes effectively deliver *E6* and *E7* knockout (*E6E7*‐KO) CRISPR/Cas9 plasmids to tumor cells, promoting gene editing that reduces HPV18 *E6* and *E7* gene expression.	Administering *E6E7*‐KO in 5‐week‐old Nude mouse xenograft (immunodeficient) via liposome NPs resulted in smaller tumor sizes and lighter weights compared to the negative control group In vivo. The high‐dose group showed the smallest tumors, and all experimental groups had significantly slower tumor growth rates than the control group, demonstrating the effectiveness of the liposome delivery method.	[69]
CRISPR/Cas9	HPV16 *E6*/*E7*	Nanoliposomes‐CRISPR/Cas9 gRNA‐HPV16 E6/E7 complex	**Size:** 145.7 ± 2.6 nm | **Zeta potential:** + 51.33 ± 1.8 mV | **Encapsulation efficiency:** 96.87 ± 0.13%	Under acidic conditions in endosomes/lysosomes, the nano‐liposome becomes positively charged, disrupting the lysosomal membrane and allowing the complex to release into the cytoplasm. This mechanism improves the escape of CRISPR/Cas9 gRNA for effective gene editing against HPV16 *E6*/*E7*.	The nano‐liposome‐CRISPR/Cas9 gRNA‐HPV16 *E6*/*E7* complex facilitated effective lysosomal escape for cytoplasmic delivery. In vivo studies in nude mouse xenograft (immunodeficient) showed a significant reduction in tumor volume in nude mice, and immunohistochemical analysis revealed decreased *E6* and *E7* expression, indicating effective inhibition of HPV oncogenes.	[64]
CRISPR/Cas9	HPV16 *E6*/*E7*	LNP	**Size:** 146.1 ± 1.7 nm | **Zeta potential:** + 52.41 ± 2.1 mV	Cationic liposomes deliver HPV gRNA to cervical cancer cells, allowing CRISPR/Cas9 to knockout oncogenes and stimulate an immune response. This enhances dendritic cell maturation, increases effector T cells, and reduces regulatory T cells and myeloid‐derived suppressor cells, thereby boosting antitumour immunity.	HPV gRNA–liposomes were found to significantly enhance autophagy and induce immunogenic cell death in SiHa cervical cancer cells, increasing the release of HMGB1 and ATP compared to controls. In vivo studies in severe combined immunodeficiency (SCID) mice demonstrated that the treatment inhibited tumor growth in hu‐PBL‐SCID mice and increased CD3 + CD8 + T cells while decreasing immunosuppressive Tregs, suggesting a promising ability to alter the tumor microenvironment.	[68]
CRISPR/Cas9	HPV16 *E7*	LNP (PEGylated liposomes)	**N/P ratio:** 16:1 | **Size:** 217 ± 13.35 nm | **PDI:** 0.39 ± 0.04 | **Zeta potential:** + 48 ± 3.12 mV	Stealth liposomes delivering Cas9 plasmids and gRNAs were used for targeted therapy in HPV16*E7*‐expressing tumors in mice. Administered via tail vein injection, this approach minimized inflammation while effectively suppressing tumor growth and accumulating in target tissues.	Stealth liposomes effectively delivered Cas9 plasmids and 16*E7*‐targeting gRNAs, leading to significant tumor control in an immunocompetent C57BL/6J mice. Continuous treatment resulted in complete tumor clearance and increased apoptosis with minimal liver toxicity. Splenic hyperplasia observed was likely a nonspecific reaction to plasmid DNA rather than inflammatory damage. Administered via tail vein injection, this approach minimized inflammation while effectively suppressing tumor growth and accumulating in target tissues.	[6]
CRISPR/Cas9	HPV16 *E6*/*E7* and HPV16 *E6*/*E7*	LNP (PEGylated liposomes)	**N/P ratio:** 16:1 | **Size:** 210 ± 10.79 nm | **PDI:** 0.41 ± 0.09 | **Zeta potential:** + 45 ± 2.71 mV	Liposomes enhance the delivery of CRISPR/Cas9 by packaging DNA and gRNAs into PEGylated lipoplexes, which protect against degradation. Designed to evade serum nucleases for hours, they maintain integrity in the bloodstream. When they reach target cells, the liposomes fuse with the cell membrane, releasing their contents into the cytoplasm.	Treatment with WT Cas9 to knockout *E6* and *E7* significantly reduced cell viability and tumor HPV growth, proving effective in suppressing cervical cancer xenografts. The liposomes successfully packaged WT Cas9 and guide RNAs, protecting them against serum nucleases for up to 6 h post‐injection. However, no detectable DNA was found in the bloodstream 4 h after injection in immunodeficient (Rag1) mice, indicating rapid clearance.	[66]
CRISPR/shRNA	HPV16 *E7*	Polymeric nanoparticle (poly (β‐amino ester) (PBAE))	**Size:** 110.2–174.7 nm | **Zeta potential:** + 13.82– + 22.28 mV	PBAE‐based nanoparticles are optimized for low toxicity and high biodegradability, making them effective for gene therapy. Their stability in the vaginal acidic environment enhances plasmid effectiveness against HPV infections and inhibits cancer cell growth.	PBAE‐based NPs showed low toxicity in C57BL/6 (immunocompetent) mice and effectively transfected cells at a 60:1 weight ratio with a 10 μg plasmid by Day 6. In HPV16‐positive cervical cancer models, PBAE/CRISPR and PBAE/shRNA NPs significantly reduced HPV16 *E7* protein levels, restored the tumor suppressor RB1, and inhibited tumor growth, resulting in treated volumes of 52 and 60 mm³, respectively.	[5]
CRISPR/Cas9	HPV16 *E7*, P16 (host cell factor)	Polymeric nanoparticle (poly (β‐amino ester) (PBAE))	**Size:** 141.2–345.9 nm | **Zeta potential:** + 15.3– + 33.0 mV	PBAE‐based nanoparticles improve the stability and delivery of plasmids for gene therapy, promoting efficient transfection into cervical cancer cells while allowing for therapeutic protein expression or CRISPR‐based inhibition of HPV‐positive cancer growth.	PBAE‐based NPs reduced the viability of SiHa and CaSki HPV16‐positive cervical cancer cells at a 60:1 mass ratio (PBAE546/plasmid) in C57BL/6 mice. In vivo studies showed significant tumor growth inhibition in SiHa tumor‐bearing mice (BALB/c‐nu nude mice) compared to the empty plasmid group. After 20 days of treatment in HPV16 transgenic mice, levels of HPV16 *E7* and P16 decreased significantly, while tumor suppressor gene RB1 levels were restored.	[70]
CRISPR/Cas9	HPV16 *E7*	Polymeric nanoparticle (PAMAM‐PBAE hyperbranched copolymer, hPPC)	**N/P ratio:** 75:1 | **Size:** 235 (hPPC1), 334 (hPPC2), 317 (PBAE) nm	The PAMAM‐PBAE hyperbranched copolymer serves as a gene delivery vehicle. Its biocompatibility and biodegradability improve cellular uptake, inhibit cervical cancer cell growth, and show low toxicity.	The hPPCs/PBAE polyplex NPs exhibited low toxicity in cervical cancer cell lines, with significant cytotoxicity above 10 μg/mL and marked cell growth inhibition at over 75 μg/mL for hPPCs/PBAE‐GFP. In nude xenograft models, hPPC1 polyplex NPs achieved a 90.3% tumor inhibition rate.	[71]
CRISPR/Cas9	HPV‐18 *E6*, HPV‐18 *E7*	The encapsulation of the chemotherapy drug Docetaxel.	**Zeta potential:** + 0.65 (DOC@DOTAP), −0.3 (E6E7), +0.3 ((DOC + E6E7)@DOTAP) mV | **Encapsulation efficiency:** 56.2%	A cationic lipid‐shell NP enhances the delivery of a CRISPR/Cas9 vector targeting HPV's *E6* and *E7* oncogenes, promoting apoptosis in HeLa cells. It also allows for the sustained release of Docetaxel when delivered to cervical cancer cells.	DOTAP is highly cytocompatibility with low toxicity up to 100 µg/mL and effectively binds to CRISPR/Cas9 vectors at a 1:2 DNA:DOTAP ratio. In vitro, (DOC + *E6E7*) induced 84.1% apoptosis in HeLa cells. In vivo, treated tumor‐bearing BALB/c nude mice showed increased survival and reduced tumor growth, with the TUNEL assay confirming enhanced apoptosis in tumor cells, indicating its therapeutic effectiveness.	[72]

Recent preclinical studies underscore the effectiveness of these nanoparticle systems in delivering CRISPR/Cas9 for targeting HPV oncogenes, particularly HPV16 *E6* and *E7*. For instance, cationic liposomes have been shown to condense and protect CRISPR/Cas9 plasmids while promoting their internalization in cervical cancer cells, ultimately leading to a significant reduction in HPV gene expression and a corresponding decrease in tumor size in animal models [6]. Furthermore, PEGylated liposomes have been designed to minimize inflammatory responses, allowing for enhanced accumulation in tumors and prolonged therapeutic effects with minimal off‐target toxicity [64]. To identify these off‐target sequences, the standard methodology for the global detection of DNA double‐stranded breaks (DSBs) induced by CRISPR RNA‐guided nucleases (RGNs) and potentially other nucleases, referred to as genome‐wide, unbiased identification of DSBs enabled by sequencing (GUIDE‐seq), is predicated on the capture of double‐stranded oligodeoxynucleotides at DSB sites [65]. Research indicates that CRISPR/Cas9 exhibits significant efficiency in the targeting of HPV E6/E7 oncogenes, with on‐target cleavage rates frequently surpassing 68%–83% in SiHa/Caski cells [66]. Furthermore, it maintains a comparatively low off‐target profile when evaluated against alternative gene‐editing methodologies such as TALENs and ZFNs. Research indicates that CRISPR/Cas9 systems engineered to target HPV16 E6/E7 exhibit minimal off‐target effects, with 0–4 off‐target sites identified [67]. This is markedly lower compared to TALENs, which present 1–36 off‐target sites, and ZFNs, which can result in dozens to hundreds of off‐target occurrences. Furthermore, on‐target activity was observed to be greater at the 72‐h mark compared to the 96‐h time point.

In addition to improving delivery efficiency, NPs can also orchestrate broader immune responses against tumors. NPs delivering CRISPR/Cas9 have demonstrated the ability to enhance dendritic cell maturation, increase effector T cell proliferation, and reduce regulatory T cell populations in the tumor microenvironment. This immunomodulatory effect not only aids in combating HPV‐associated tumors but also serves to prime the immune system for enhanced cancer surveillance [68].

Moreover, cutting‐edge work with polymeric nanoparticles or cationic polymers like poly (β‐amino ester) (PBAE) shows promise for achieving effective plasmid delivery in acidic environments, which is particularly relevant for targeting HPV infections. PBAE‐based NPs have successfully transfected cervical cancer cells with CRISPR constructs, resulting in significant protein level reductions and tumor volume decreases in preclinical models [69]. Collectively, these developments in NP‐based gene therapy highlight a transformative approach to treating HPV‐related cancers, moving towards safer and more efficient therapeutic strategies with the potential for future clinical translation.

#### 4.1.2 SiRNA

The in vivo delivery of siRNA presents a series of complex challenges that hinder its therapeutic applications. One of the major barriers is the instability of siRNA when in the bloodstream, where nucleases rapidly degrade these molecules, and they are cleared by renal filtration due to their small size and hydrophilic nature [73]. Furthermore, struggles in crossing the cellular membrane are presented for negatively charged siRNA with the cell membranes hydrophobic lipid bilayer, necessitating the use of vector‐mediated delivery such as LNPs and polymer‐based systems to facilitate cellular uptake [74]. However, these vectors are presented with whole new array of limitations, including low targeting specificity, off‐target accumulation, and potential induction of immune responses [75]. Successful endosomal escape following cellular uptake is yet another critical hurdle siRNA clinical translation faces, as siRNA must reach the cytoplasm to engage the RISC effectively [76]. Efficient and targeted delivery of siRNA to specific tissues are also faced with immunological obstacles, primarily avoiding rapid clearance by the MPS.

Combining all these challenges, ensuring the stability of siRNA within the systemic circulation and protecting it from enzymatic degradation also remains difficult. To overcome these multifaceted hurdles, innovative delivery strategies, preclinical evaluation and optimization of formulation parameters must be addressed.

In relation to cervical cancer treatment, systemic administration of *siE6/E7* still remains a major challenge with the negatively charged macromolecular structure of the siRNA preventing passive diffusion across the cell membrane and into the cytoplasm as well as renal filtration and enzymatic degradation [73, 74, 77]. Further research is needed to explore more efficient, safe, and targeted delivery systems to translate siRNA therapies into clinical practice. Pathways in overcoming these challenges are being achieved in the extensive development of delivery vehicles to protect siRNA during circulation and accelerate the tumor tissue accumulation as well as siRNA uptake by cancer cells, through incorporation of cationic lipids, peptides and polymers [73, 74, 77] (see Table 2).

**Table 2:** Overview of siRNA utilizing Trojan horse‐like nanoparticles for HPV in preclinical trials.

SiRNA	Target gene	Nanoparticle		Mechanism of nanoparticles	Therapeutic efficacy	Ref.
		Material	Properties			
si16E6/E7si18E6/E7	*HPV E6* and *E7*	Inorganic nanoparticles Polyion Complex (PIC) micelle	**Si16E6/E7:** Size 35 ± 0.3 nm | PDI 0.11 ± 0.00 | Zeta −0.6 ± 0.1 mV **Si18E6/E7:** Size 36 ± 0.1 nm | PDI 0.10 ± 0.01 | Zeta −1.1 ± 0.2 mV	The PIC micelles feature a biocompatible poly (ethylene glycol) (PEG) outer shell, which helps maximize nonspecific protein absorption. To address potential challenges with cellular uptake by target cells, poly(l‐lysine) (PLL) was incorporated, providing thiol‐containing functional groups that facilitate enhanced internalization.	Intravenous administration of *si16E6/E7*‐loaded PIC micelles, at siRNA concentration of 20 nM, significantly inhibited the proliferation of HPV‐associated cervical cancer cells by effectively silencing *E6* and *E7* oncogenes in *SiHa* and *HeLa* tumor models. Subcutaneous xenograft models were used. Biodistribution analysis using fluorescence imaging demonstrated efficient accumulation of the siRNA‐loaded PIC micelles within the tumor site following systemic delivery. Furthermore, histological staining of tissue samples revealed no evidence of apoptosis in kidney tissues, suggesting that the PIC micelles did not induce notable tissue toxicity.	[77]
si18E7‐674	*HPV‐18* containing HeLa cells	LNP (PEGylated liposomes)	**N/P ratio 2:** Size ~260 nm | Zeta potential ~−11 mV	PEGylated liposomes are spherical, hollow phospholipid NPs coated with a polyethylene glycol (PEG) layer that helps shield them from immune detection and rapid clearance from the bloodstream. PEGylation has been shown to prolong circulation time, reduce immunogenicity, and enhance tumor accumulation following intravenous administration	The liposomal vehicle facilitated targeted cellular uptake and improved bioavailability of the therapeutic agent at the tumor site, with improved tumor accumulation of 10–20‐fold greater than naked NPs. The viral‐specific gene silencing reduced off‐target effects and achieved more than 95% protein silencing in vivo. An increase in gene silencing was observed with higher N:P (nitrogen:phosphate) ratios, suggesting that PEI complexation enhanced delivery efficiency—potentially through improved siRNA release and/or endosomal escape. Notably, PEI‐mediated endosomal escape may also help reduce immunogenicity, as endosomal acidification is a key trigger for siRNA‐induced interferon and cytokine responses. Moreover, PEGylated *si18E6‐674*‐loaded nanocarriers, loaded with 2.5 nM of siRNA, induced greater apoptosis in HPV‐18‐positive *HeLa* cells.	[78]
			**N/P ratio 4:** Size ~240 nm | Zeta potential ~−13 mV			
			**N/P ratio 6:** Size ~180 nm | Zeta potential ~+3 mV			
			**N/P ratio 8:** Size 200 nm | Zeta potential ~+12 mV			
Anti‐E7 siRNA	*HPV18‐E7*	siRNA/PTX co‐loaded polymeric nanoparticles camouflaged with Hela cell membranes (Si/PNP@HeLa)	**Size:** 194.1 nm |	Paclitaxel (PTX) and siRNA targeting *HPV18‐E7* were co‐encapsulated within a PLGA core, which was then coated with membranes derived from *HeLa* cancer cells to create a biomimetic, homotypic targeting NP. This design leveraged the tumor‐homing properties and high biocompatibility conferred by the preserved *HeLa* cell membrane antigens and structural components on the NP surface. As a result, the *Si/PNPs@HeLa* NPs exhibited enhanced tumor‐specific accumulation and increased cellular uptake.	Beyond restoring the expression of the tumor‐suppressor protein Rb, siRNA targeting *E7* also counteracts PTX resistance by inhibiting activation of the AKT signaling pathway, resulting in a synergistic enhancement of antitumour efficacy in cervical cancer. The gene silencing efficiency was notably improved following HeLa cell membrane coating, as the biomimetic shell facilitated greater NP accumulation and improved immune escape ability—threefold higher than bare nanoparticles. *Si/PNP@HeLa* NPs loaded with *anti‐E7 siRNA* effectively reduced cell viability and suppressed tumor growth in vivo with tumor volume inhibiting rates of 83.6% and no side effects in major organs. A subcutaneous HeLa xenograft model was used.	[63]
			**PDI:** 0.143 |			
			**Zeta potential:** −30 mV |			
			**Encapsulation efficiency:** 88.4 ± 0.22%			
ENB101‐LNP	*HPV16 E6* and *E7*	Ionizable lipid nanoparticles encapsulating siRNA against *E6/E7* of *HPV16* combined with cisplatin	No data available	The lipid components of the NP play a critical role in enhancing both delivery and targeting efficiency. Cholesterol contributes to the structural stability of LNPs by modulating membrane integrity and rigidity. PEG further improves NP stability, delivery, tolerability, and biodistribution. Cisplatin, a platinum‐based chemotherapeutic agent, is widely used in the treatment of various cancers. It exerts its anticancer effects by binding to and damaging DNA within cancer cells, thereby inhibiting their ability to grow and divide.	*ENB101‐LNP* and cisplatin work synergistically to inhibit *CaSki* cell growth. The combination therapy decreases *HPV16 E6/E7* mRNA levels while increasing the expression of *p21* mRNA, as well as *p53*, *p21*, and HLA Class I proteins. Tumor inhibition rates of the high‐dose combination delivery were 68.8%. RT‐PCR analysis demonstrated up to 80% knockdown of *HPV16 E6/E7* in the *ENB11‐*LNP‐treated groups. This NP formulation significantly reduced cell viability, inhibited tumor growth, and promoted apoptosis. A xenograft model was made using CaSki cells in BALB/c nude mice.	[79]

### 4.2 Clinical Trials

Currently, there is only one known clinical trial investigating the application of CRISPR/Cas9 for the disruption of HPV Types 16 and 18 *E6*/*E7* (Clinical Trial No. NCT03057912). This trial, which is in Phase 1, is sponsored by the First Affiliated Hospital, Sun Yat‐Sen University in China (Table 3) and is designed to evaluate safety and efficacy. The trial details indicate that the Cas9 protein and guide RNAs are encoded on a plasmid, which is then delivered to cervical epithelial cells through a locally applied topical gel designed for HPV‐infected cervices [80]. As of 2026, the status of the data collection remains unknown, as the trial commenced in 2018 and was anticipated to conclude in 2019 and the sponsor has yet to disseminate outcome data to the public.

**Table 3:** Overview of gene‐targeted therapies for HPV in clinical trials.

Gene tool	Target gene	Drug delivery	Carrier	Phase	Sponsors	Clinical trial no.
CRISPR/Cas9	HPV16/18 *E6*/*E7*	Topical gel drug	N/A	I (2018–2019)	Sun Yat‐Sen University, China	NCT03057912

## 5 Challenges

### 5.1 Clinical Translation

Despite promising preclinical outcomes, the clinical translation of siRNA‐loaded and CRISPR/Cas9‐loaded NPs faces significant challenges. Cellular mitosis results in halving siRNA concentration with every division, as siRNA molecules are passively distributed between daughter cells and not replicated like genomic DNA. This dilution leads to reduced therapeutic concentration, particularly affecting sustained gene silencing in rapidly dividing tissues such as HPV‐related tumors. There is a need for strategies to prolong siRNA stability or enable continued dosing for potent cytotoxic effects. Fortunately, this issue does not affect CRISPR/Cas9 therapies, as they edit the DNA of cells and are not duplicated during mitosis [81].

Onset of action time is another consideration. The time it takes for siRNA delivered by nanoparticles to reach tumor sites varies based on NP design and tumor location; significant accumulation can generally be observed at 4–6 h post‐intravenous injection, with a study showing 70%–80% of injected siRNA/g in the tumor site within 4 h [82]. Additionally, NP formulations often encounter scalability, reproducibility, and regulatory compliance issues, including immunogenicity and potential toxicity [76]. Endosomal escape of therapeutic cargo before degradation by lysosomes is crucial and is typically achieved using positively charged moieties or fusogenic helper [83]. These approaches are utilized in both siRNA and CRISPR/Cas9 NP preclinical trials (see Tables 1 and 2).

### 5.2 Safety Concerns

Trojan horse NPs hold great promise for targeted drug delivery and precision medicine, but their safety remains a critical concern. A major issue is the potential to trigger unintended immune responses, as the body may recognize these engineered particles as foreign invaders, leading to inflammation or hypersensitivity. Additionally, materials used in constructing NPs, such as certain metals or synthetic polymers, can accumulate in tissues and cause toxicity over time. These effects may be dose‐dependent and influenced by particle size, surface chemistry, and biodegradability [84, 85]. Comprehensive preclinical evaluation and long‐term toxicity studies are essential to ensure that Trojan horse nanoparticles can be safely used in clinical applications. All future clinical trials must adhere compliance with Part 58 (Good Laboratory Practice for Nonclinical Laboratory Studies) of Title 21 of the Code of Federal Regulations (GFP CFR 21) with respect to toxicology studies.

In the context of cervical cancer, often caused by persistent infection with high‐risk HPV, Trojan horse NPs offer a promising approach to deliver antiviral agents or immunotherapies directly to infected or cancerous cells. This can be achieved through tailored engineering to selectively bind to HPV‐infected cells using ligands or antibodies that recognize virus‐specific or tumor‐associated markers. However, safety concerns remain regarding the long‐term interaction of these NPs with healthy reproductive tissues as investigated in sarcoma xenograft models [86]. The mucosal environment presents additional challenges, as NPs must avoid disrupting the natural microbiota or causing local irritation. Any immunomodulatory strategies must also be carefully evaluated to prevent overactivation of immune responses, which could damage surrounding tissues or impact fertility [86]. Thus, while Trojan horse NPs represent a powerful strategy against HPV‐driven cervical cancer, rigorous safety assessments are essential for their clinical translation.

### 5.3 Global Health Challenges

From an economical and promotional perspective, siRNA‐loaded NP therapeutics offer a highly targeted approach to disease treatment, particularly for unmet medical needs such as certain cancers, genetic disorders, and viral infections. Economic opportunities face challenges with the development and commercialization processes involving high costs due to complexities of nanoparticle formulation [87], regulatory approval, and manufacturing scale‐up. In high socioeconomic countries, infections are prevalent, yet 85% of cervical cancer deaths occur in LMICs [88]. Many in these regions may not be able to afford NP treatments, like PEGylated Liposomal Doxorubicin (PLD), which costs at least US$873 for a 20 mg vial [89]. Some cheaper formulations lack efficiency, and evolving cancers necessitate multifunctional NPs, increasing complexity and costs [87]. Despite these barriers, high returns may exist, with orphan drug status offering incentives for pharmaceutical companies. Successfully translating these therapies into clinical applications while remaining commercially sustainable requires strategic collaborations, public‐private funding, and early regulatory engagement. To enhance accessibility, scalable manufacturing, equitable pricing, and international collaborations are essential for addressing healthcare disparities in LMICs [90]. For implementation in LMICs, it would be advantageous to explore a range of feasible delivery modalities including topical vaginal creams or gels, intravaginal rings, mucoadhesive patches, lyophilized powders and oral formulations, to maximize accessibility and scalability of emerging therapeutics for HPV induced cancers.

### 5.4 Alternative Delivery Methods

RNA‐based therapies targeting HPV are advancing globally, with delivery systems playing a crucial role in their efficacy. Successful encapsulation of both mRNA and siRNA affording protection against degradation has made LNPs a prominent delivery method to facilitate cellular uptake. For instance, HPV16 RNA‐LPX utilizes LNPs to deliver mRNA encoding HPV antigens directly to dendritic cells, enhancing immune responses [91]. Similarly, PEGylated lipid particles have demonstrated a 50% reduction in tumor size in HPV‐positive cervical cancer mouse models [78]. These systems improve transfection efficiency and biodistribution, making them effective in preclinical settings.

Despite these advancements, challenges persist in translating these strategies to clinical practice. siRNA therapies face hurdles such as instability in the bloodstream, off‐target effects, and inefficient delivery to tumor sites. Nanocarriers such as chitosan‐based NPs have shown in vitro promise, but their clinical application remains limited due to stability and toxicity issues [92]. Furthermore, the lack of appropriate preclinical models that fully replicate HPV‐associated cancers hinders the assessment of these therapies’ efficacy and safety [93]. Addressing these challenges is essential for successful clinical implementation of RNA‐based HPV therapies.

In the realm of targeted therapies against HPV, both viral and non‐viral vectors offer distinct advantages and limitations. Viral vectors, such as adenovirus and lentivirus, provide prolonged gene expression and high transfection rates, which are beneficial for therapeutic applications requiring sustained activity [94]. Their mechanisms for cell entry make them effective in targeting both dividing and non‐dividing cells [95]. For large scale production, vectors can be propagated in suspension culture bioreactors using a continuous cell line, and can then be purified by ion exchange and gel filtration chromatography as seen in poxvirus vectors [96]. However, their use is limited by immunogenicity, the risk of insertional mutagenesis, and challenges sourcing lipid and polymer materials for large‐scale manufacturing [97]. Additionally, conveying to patients the injection of live viruses or genetically modified organisms (GMOs) may intimidate laypeople and discourage them from accepting this therapy, impacting manufacturing costs and driving developers toward more complex non‐viral alternatives.

Non‐viral delivery systems, including LNPs, polymer‐based carriers, and exosomes, offer improved safety profiles, reduce immunogenicity, and allow customization and easier production [98]. These systems are more suitable for repeated dosing and present a lower risk of genomic integration [99]. Nonetheless, non‐viral vectors often suffer from lower transfection efficiency and rapid clearance from the body, compromising their therapeutic impact [100]. Balancing these factors is crucial for the future development of effective RNA‐based therapies against HPV.

## 6 Future Perspectives

The future of gene‐targeted therapies for HPV infections is promising, especially with innovative techniques like RNAi and CRISPR/Cas delivered via Trojan horse NPs. For example, co‐encapsulating paclitaxel with radioluminescent NPs in biodegradable polymers may improve radiotherapy effectiveness for advanced solid tumors, offering significant cancer cell lethality with reduced systemic toxicity [101].

The concept of nanovaccines further amplifies the potential of Trojan horse NPs. By integrating therapeutic and prophylactic effects, these nanovaccines can elicit robust immune responses while simultaneously delivering targeted therapies. Clinical research indicates that therapeutic vaccines focused on specific HPV antigens can induce cytotoxic T cell responses capable of eliminating HPV‐positive cells [102]. The use of NPs as carriers for these nanovaccines enhances their immunogenic profiles by improving cellular uptake and presentation, thereby fostering systemic immunity against HPV‐associated cancers [103].

As targeted therapies evolve alongside prophylactic vaccination strategies, increasing access to and uptake of HPV vaccines across diverse populations remains essential. Significant barriers, including socio‐cultural factors, misinformation, and inadequate healthcare provider recommendations, pose challenges to the successful implementation of vaccination programs [104]. Future initiatives must not only focus on developing gene‐targeted therapies but also implement public health strategies that promote education and awareness around HPV vaccination, ensuring that innovative treatments integrate effectively into broader preventive measures against HPV‐related malignancies [105].

Current studies reveal a notable interplay between HPV status and treatment response in head and neck cancers. HPV‐positive cancers often display distinct biological characteristics that influence their response to conventional therapies such as chemotherapy and radiotherapy. Furthermore, gene‐based approaches could enhance therapeutic efficacy by tailoring treatment regimens for this patient population [106]. Research highlights that clinical outcomes for patients with HPV‐positive tumors often surpass those of HPV‐negative tumors, underscoring the importance of personalized treatment strategies [107].

Additionally, exploring metabolic and immunological profiles in HPV‐driven malignancies has revealed significant potential in integrating immune checkpoint inhibitors with gene‐targeted therapies. The combination of RNAi or CRISPR methodologies with immune checkpoint blockade may activate anti‐tumor immunity while addressing immune evasion mechanisms in HPV‐positive tumors [108]. Studies indicate that HPV‐positive cancers present specific immune exclusion patterns, and disrupting these via targeted therapeutic interventions could enhance patient responses to existing immunotherapies [109].

Understanding the tumor microenvironment of HPV‐related cancers, which harbors unique cellular and molecular properties, is also crucial. Investigating the interplay between immune responses and gene‐targeted therapies will help the development of effective treatment plans that minimize adverse effects on healthy tissues [110].

A comprehensive strategy that includes ongoing basic and translational research, clinical trials, and community health initiatives will be pivotal in maximizing the potential of these therapeutic approaches. Over the next decade, we expect to see major improvements in how we treat HPV. These advancements will not only help more patients survive but also improve their quality of life. Additionally, the emergence of novel biomarkers and genetic profiling methods will improve the identification of patient populations most likely to benefit from individualized therapies [111].

## 7 Conclusion

HPV is a leading sexually transmitted infection and a major contributor to cervical cancer. Global disparities in vaccination access exacerbate the public health challenge, particularly in LMICs. Current treatment options, mainly surgical and chemoradiation, have limited success, emphasizing the need for innovative therapeutic strategies. This review, therefore, highlights the potential of gene‐targeted therapies, including CRISPR/Cas systems and siRNA technologies, to address HPV‐related diseases. Additionally, the use of Trojan horse NPs enhances therapeutic delivery by targeting HPV‐infected cells while reducing systemic toxicity. By leveraging advancements in nanotechnology and gene therapy, this approach promises to transform treatment paradigms for HPV‐associated malignancies. The review aims to present these innovative strategies and their implications for improving outcomes in patients suffering from HPV‐related conditions.

## Author Contributions

**Trairong Chokwassanasakulkit:** writing – review and editing, writing – original draft, visualization, conceptualization. **Vindya Ranasinghe:** writing – review and editing, writing – original draft, designing the figure. **Erin Woods:** writing – review and editing, writing – original draft, visualization. **Linh Q. Nguyen:** writing – review and editing, writing – original draft. **Nigel A. J. McMillan:** writing – review and editing, writing – original draft, supervision, project administration, funding acquisition, conceptualization.

## Ethics Statement

This article is a review of studies that have been previously published and does not involve any human participants or animals that were performed by the authors. Consequently, informed consent and ethical approval were not required.

## Conflicts of Interest

The authors declare no conflicts of interest.

## Data Availability

Data sharing not applicable to this article as no datasets were generated or analysed during the current study.
